# Supplementary material for: The GlyGly-CTERM domain functions as an independent motif that targets proteins to rhombosortase in Vibrio cholerae
Source: J Bacteriol. 2026 Jun 12;208(7):e00146-26. doi: 10.1128/jb.00146-26 (PMC13393428; doi:10.1128/jb.00146-26)
Supplement: Supplemental Figures and Tables — Figures S1 to S6 and Tables S1 to S4. [file jb.00146-26-s0001.pdf]

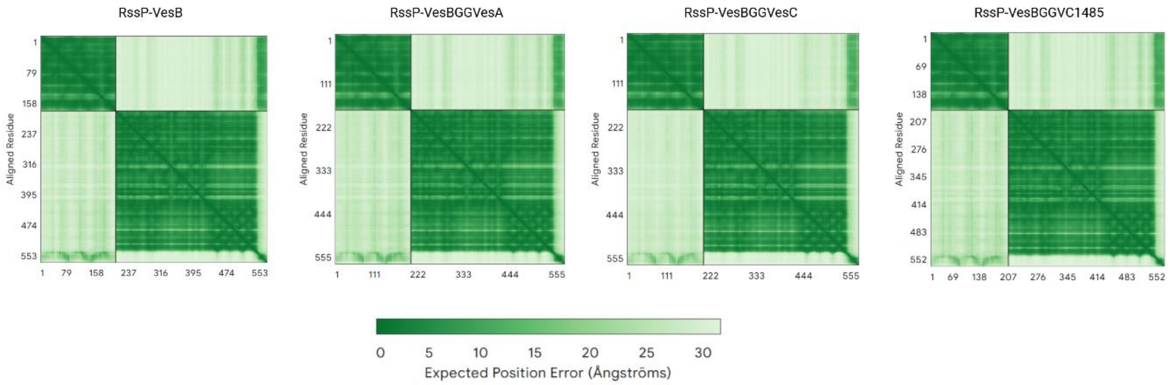

Supplemental Figure 1. Alignment error for RssP and VesB chimeric protein interaction. The primary amino acid sequences of RssP and VesB or VesB chimera were used to predict the interaction using AlphaFold3. Position error shown.

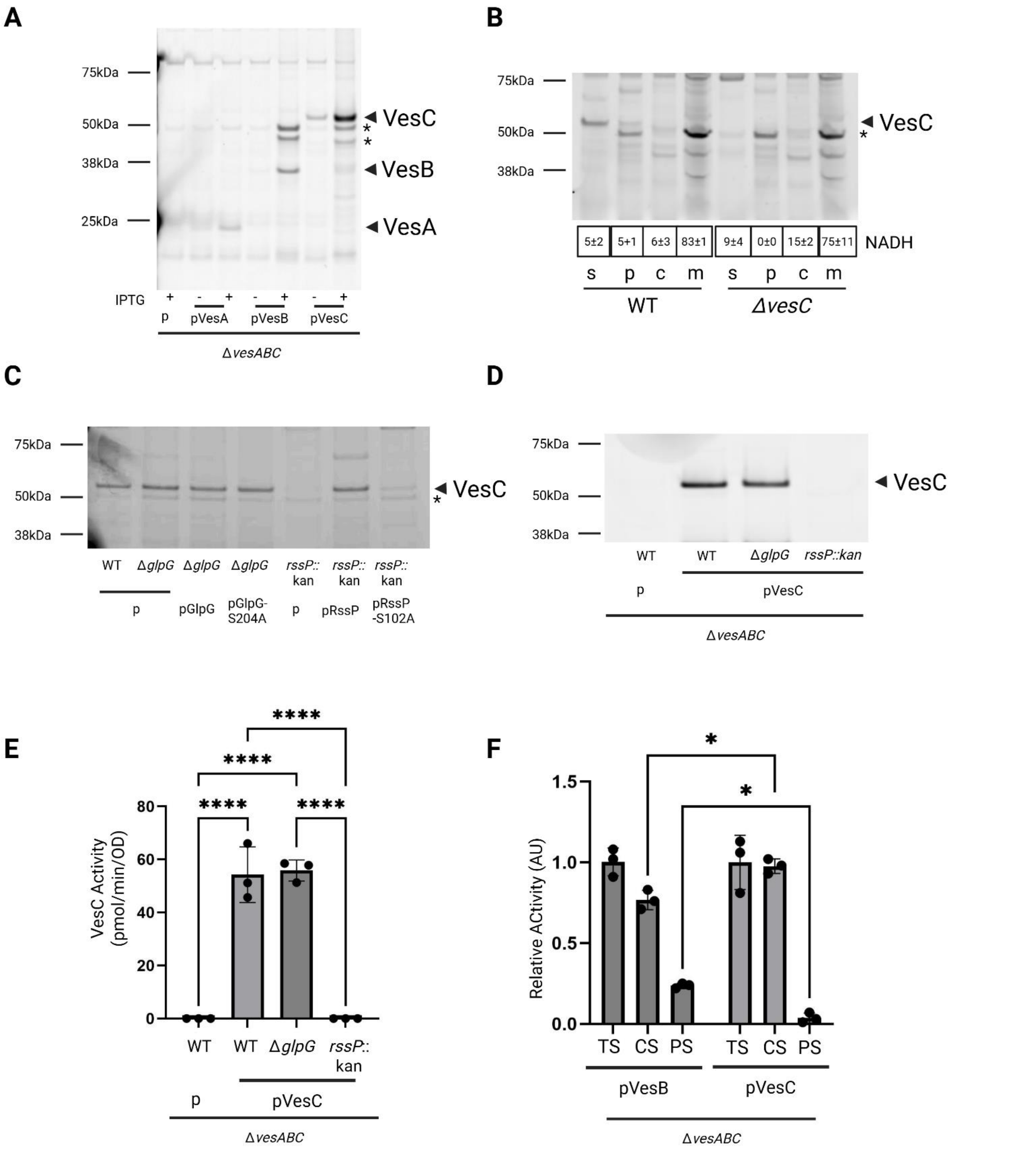

Sup. Figure 2. VesC is secreted into the extracellular space in an RssP-dependent manner. A. Supernatants from overnight cultures of WT strain N16961 containing empty vector or plasmids expressing indicated Ves protease were isolated, incubated with ActivX serine hydrolase probe, before separation by SDS-PAGE and fluorescent gel imaging. Where indicated, IPTG was used at 50 μM for expression of VesA and VesB and 10 μM for VesC. VesA, VesB, and VesC and molecular weight markers are indicated. Representative gel is shown (n=2). Asterisks show non-specific bands. **B.** Log phase cultures of WT or Δ*vesC* mutant cells were fractionated into supernatant (s), periplasmic (p), cytoplasmic (c), and membrane (m) fractions, incubated with ActivX serine hydrolase probe, before separation by SDS-PAGE and fluorescent gel imaging. As a control for membrane isolation, NADH dehydrogenase activity was monitored by measuring decrease in absorbance at 340

nm over time. Average ± standard deviation from technical duplicates is shown. Molecular weight markers and VesC are indicated. Non-specific band is indicated with asterisk. Representative gel is shown (n=2). **C.** Supernatants from overnight cultures of WT and indicated rhomboid mutants containing empty vector or complemented with indicated plasmid were isolated, incubated with ActivX serine hydrolase probe before separation by SDS-PAGE and fluorescent gel imaging. VesC and molecular weight markers are indicated. Representative gel is shown (n=2). **D.** Supernatants from WT and indicated rhomboid mutants containing empty vector or ectopically overexpressing (10 µM IPTG) VesC were processed and visualized as in **C**. VesC and molecular weight markers are indicated. Representative gel is shown (n=2). **E.** Supernatants from **D** were assessed for serine protease activity against the fluorogenic peptide Boc-Gln-Ala-Arg-AMC. Data represent mean ± SD of n = 3 experiments in technical triplicate with Tukeys multiple comparison test with **** representing $p \leq 0.0001$. Only statistically significant comparisons are shown. **F.** Supernatants from overnight cultures of ΔvesABC mutant strain ectopically overexpressing (10 µM IPTG) VesB or VesC were isolated (S), separated into cleared supernatant (CS) and pelleted supernatant (PS) before assessing for serine protease activity against the fluorogenic peptide Boc-Gln-Ala-Arg-AMC. Activity is expressed relative to the total supernatant (S) activity. Data represent mean ± SD of n = 3 experiments in technical triplicate with Tukeys multiple comparison test with * representing $p<0.05$.

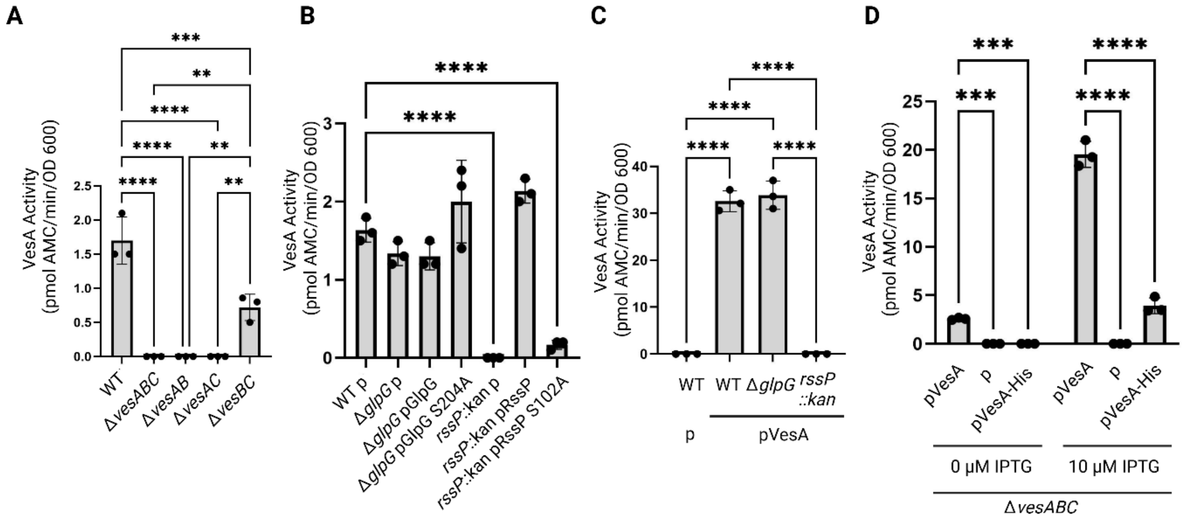

Sup. Figure 3. VesA requires RssP and an intact GlyGly-CTERM for efficient activation. A. Supernatants from overnight cultures of WT and indicated mutant strains were isolated and assessed for serine protease activity using the fluorogenic peptide substrate Suc-Leu-Leu-Val-Tyr-AMC. Data represent mean ± SD of n = 3 experiments in technical triplicate with Tukeys multiple comparison test with ** representing p<0.005, *** p<0.0005 and **** p≤ 0.0001. Only significant differences are shown. **B.** Supernatants from overnight cultures of WT and indicated rhomboid mutant strains ectopically complemented with WT rhomboid protease or catalytic mutant were isolated and assessed for serine protease activity using the fluorogenic peptide substrate Suc-Leu-Leu-Val-Tyr-AMC. Data represent mean ± SD of n = 3 experiments in technical triplicate with Dunnett's multiple comparison test with **** representing p≤0.0001. Only significant differences are shown. **C.** Supernatants from overnight cultures of WT and indicated mutant strain containing empty vector (p) or overexpressing (50 µM IPTG) plasmids encoding WT VesA were isolated and assessed for serine protease activity using the fluorogenic peptide substrate Suc-Leu-Leu-Val-Tyr-AMC. Data represents ± SD of n = 3 experiments in technical triplicate with Tukeys multiple comparison test with *** representing p<0.0005 and **** p≤0.0001. Only significant differences are shown. **D.** Supernatants from overnight cultures of the mutant strain Δ*vesABC* containing empty vector (p) or plasmids encoding WT VesA or indicated VesA mutant supplemented with indicated IPTG concentration were isolated and assessed for serine protease activity using the fluorogenic peptide substrate Suc-Leu-Leu-Val-Tyr-AMC. Data represent mean ± SD of n = 3 experiments in technical triplicate with Sidak's multiple comparison test with *** representing p<0.0005 and **** p≤ 0.0001. Only significant differences are shown.

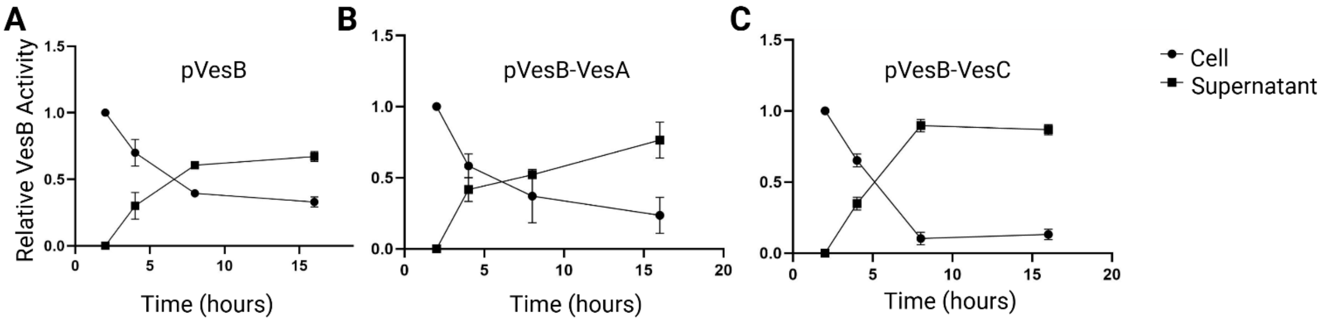

Sup. Figure 4. The GlyGly-CTERM and disordered region from VesC transiently associates the passenger protein with the cell fraction. Mutant strain Δ*vesABC* expressing VesB (**A**), VesB-VesA (**B**), or VesB-VesC (**C**) chimera were grown for 16 hours. At indicated timepoints, samples were taken and separated into cell and supernatant fractions before they were assessed for serine protease activity against the fluorogenic peptide Boc-Gln-Ala-Arg-AMC. Relative activity was calculated by taking the cell and supernatant activity, respectively, and diving by the total activity across the cell and supernatant fractions at the indicated time point. Data represent mean ± SD of n = 3 experiments in technical triplicate.

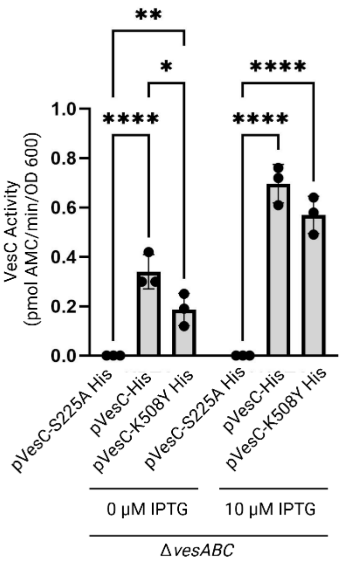

Sup. Figure 5. VesC-K508Y His6 is active. Culture supernatants from the Δ*vesABC* mutant strain ectopically expressing different VesC-His6 variants in the absence and presence of IPTG were isolated and assessed for serine protease activity against the fluorogenic peptide Boc-Gln-Ala-Arg-AMC. Data represent mean ± SD of *n* = 3 experiments in technical triplicate with Sidak post hoc analysis with * representing p≤0.05 **p≤0.005, and ****p≤0.0001. Only statistically significant comparisons are shown.

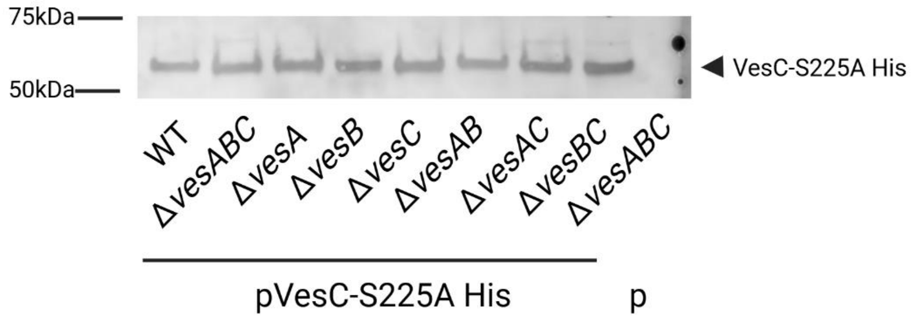

Sup. Figure 6. VesC-His is not processed in trans. Culture supernatants from indicated mutant strains containing empty vector or encoding catalytically inactive VesC-His6 were isolated, run on SDS-PAGE, transferred to nitrocellulose, and blotted against anti-His6 antibodies. VesC species and molecular weight markers are indicated. Representative blot is shown (n=2).

Table S1. Proteins significantly enriched in the WT or *rssP*::kan membranes.

Proteins enriched in WT membranes compared to *rssP*::kan	Proteins enriched in *rssP*::kan membranes compared to WT
Q9KR98	Q9KVA5
Q9KVH1	Q9KQH8
Q9KNA1	P32557
Q9KTW0	H9L4T3
Q9KNX0	Q9KPM0
Q9KN29	Q9KQS6
Q9KLE3	Q9KPV3
Q9KVI9	Q9KTZ4
Q9KM21	Q9KS38
Q9KRZ8	Q9KLZ1
Q9KTJ2	Q9KLX5
Q9KQ17	Q9KUN2
Q9KTD0	Q9KRB6
Q9KTD8	Q9KMZ5
Q9KQZ3	Q9KS46
Q9KUK6	Q9KNM3
P0C6C7	Q9KPQ4
Q9KRL5	Q9KTD4
Q9KS75	O30862
Q9KKR0	Q9KVN3
Q9KM30	Q9KQM1
Q9KLK9	Q9KTM7
Q56652	Q9KSY0
Q9KMF4	Q9KUQ3
Q9KRL6	Q9KVD7
Q9KM13	Q9KQ38
P29485	Q9KRG4
Q9KSY8	Q9KNV2
Q9KQV0	Q9KRS3
Q9KUM4	Q9KQV6
Q9KUW0	Q9KTD7
Q9KRJ7	Q9KNQ0
Q9KVE6	Q9KUU6
Q9KT74	Q9KU92
Q9KSK8	Q9KPL2
Q9KSV6	Q9KQ39
	Q9KSU6
	Q9KM00
	Q9KRS1
	Q9KT52

	Q9KV92
	P23247
	Q9KNC3
	Q9KRC3
	Q9KSX4
	Q9KT55
	Q9KPT9
	Q9KL03
	Q9KP88
	Q9KRX5
	Q9KUD1

Table S2. Proteins significantly enriched in the WT or Δ*glpG* membranes.

Proteins enriched in WT membranes compared to Δ*glpG*	Proteins enriched in Δ*glpG* membranes compared to WT
Q9KLA9	Q9KVD5
O07898	Q9KSS0

Table S3. Proteins significantly enriched in the WT or *rssP*::kan supernatants

Proteins enriched in WT supernatants compared to *rssP*::kan	Proteins enriched in *rssP*::kan supernatants compared to WT
Q9KMX1	Q9KVI8
Q9KVI9	Q9KL48
Q9KQY0	Q9KL36
Q9KKN3	Q9KLY1
Q9KL84	Q9KRN3
Q9KLK5	Q9KPT3
Q9KM21	Q9KP73
Q9KTD8	Q9KPY2
Q9KTD0	Q9KT71
Q9KRJ1	P08038
Q9KU21	Q9KL61
Q9KLK9	Q9KV29
Q9KL42	Q9KTE6
Q9KQZ3	Q9KQG2
Q56652	Q9KVG0
Q9KUE0	Q9KVS2
Q9KQ01	Q9KTZ6
Q9KKS3	Q9KRY9
Q9KQ00	Q9KQX2
Q9KPR1	Q9KNS1
Q9KSV2	Q9KQI4
Q9KTF4	Q9KL62
P0C6Q6	Q9KQP8
Q9KV26	Q9KV26
Q9KPW0	Q9KP44
Q9KQI7	Q9KP17
P0C6C4	Q9KTY5
Q9KQL0	Q9KPB5
Q9KR13	O32445
	Q9KUI4
	Q9KPV4
	Q9KT67
	Q9KNV5
	Q9KU57
	O68844
	P32557
	Q9KSB3
	Q9KM75
	Q9KSG3
	Q9KUE5
	Q9KKS5

	Q9KQ52
	Q9KPW1
	Q9KTB7
	Q9KLX7
	Q9KLN2
	Q9KSS3
	Q9KUY6
	Q9KQT2
	Q9KPK7
	Q9KQH2
	Q9KVM1
	Q9KRY7
	Q9KNI7
	Q9KTT2
	Q9KMV6
	Q9KPP6
	Q9KL24
	Q9KVA5
	P0C6C8
	Q9KTM7
	Q9KP64
	Q9KUG4
	Q9KLK6
	Q9KUG5
	Q9KNT2
	Q9KNR1
	Q9KUU3
	Q9KU60
	Q9KQ38
	Q9KPJ1
	Q9KLC6
	Q9KPA1
	Q9KL30
	Q9KPV1
	Q9KLD2
	Q9KPF0
	Q9KV62
	H9L4S8
	Q9KQH9
	Q9KV14
	Q9KLT9
	Q9KLN7
	Q9KKQ2
	Q9KLE1
	Q9KPG5
	Q9KQP2
	Q9KMX4

	Q9KP37
	Q9KPE6
	Q9KSC1
	Q9KQC8
	Q9KV27
	Q9KM23
	Q9F854
	Q9KPP4
	Q9KRF0
	Q9KNL8
	Q9KQU9
	Q9KPJ0
	Q9KNM4
	Q9KVN3
	Q9KT69
	Q9KRB0
	Q9KPK3
	Q9KUN7
	Q9KKQ3
	Q9KRX5
	Q9KUW5
	Q9KRF7
	Q9KSC0
	Q9KLH7
	Q9KSN0
	Q9KPB2
	Q9KTD7
	Q9KNH7
	Q9KUP7
	Q9KMR6
	Q9KSS4
	Q9KTY2
	Q9KVN8
	Q9KMZ7
	Q9KSI6
	Q9KTY0
	Q9KLX4
	Q9KRP0
	Q9KQH5
	P50529
	Q9KQ14
	Q9K2P7
	Q9KTY4
	Q9KP79
	Q9KNU2
	Q9KSL7
	Q9KTC2

	Q9KRH0
	Q9KSM0

Table S4. Strains and plasmids used in this study.

Plasmid/strain	Description	Fwd Primer 5' to 3'	Rev Primer 5' to 3'
N16961	El tor, *Vibrio cholerae* strain		
NΔ*glpG* (1)	in frame deletion of *glpG* in N16961		
NΔ*epsD* (2)	in frame replacement of *epsD* with kanamycin cassette		
N *rssP*::kan (1)	Insertion of kanamycin cassette into *rssP*		
NΔ*glpG* *rssP*::kan (1)	*glpG* and *rssP* inactivated		
NΔ*hapA*	Kind gift from Dr. Richard Finkelstein		
NΔ*vesABC* (3)	*vesA, B, C* mutant		
NΔ*vesAB* (3)	*vesA, B* mutant		
NΔ*vesBC* (3)	*vesB, C* mutant		
NΔ*vesAC* (3)	*vesA, C* mutant		
NΔ*vesC* (3)	in frame deletion of *vesC*		
NΔ*vesA* (3)	In frame replacement of *vesA* with Cm cassette		
NΔ*vesB* (3)	In frame replacement of *vesB* with Kan cassette		
pRK2013 (4)	Helper Strain		
SY327λpir (5)	Helper strain		
pMMB67 (7)	Expression vector (7)		
pMMB960 (8)	pMMB67 with Kan^r instead of Amp^r		
pCVD442 (5)	Suicide vector (5)		
pRssP (1)	RssP expressed from pMMB67		
pRssP S102A (1)	Catalytic serine mutated to alanine		
pGlpG (9)	GlpG expressed from pMMB67		
pGlpG S204A	Catalytic serine mutated to alanine		

pHapA (10)	HapA expression from pMMB66EH		
pHapA-GG	Used pHapA and pVesB as template	GGCAAAAGGCGAGTCCAAGCGTAACGTCACACCAGA	TTACGCTTGGACTCGCCTTTTGCCCCAAGTGCCTCT
pβ-lac (2)	B-lactamase gene from pMMB67EH		
pβ-lac-IG	Used pβ-lac and GlyGly-CTERM VesB mutant as template, pMMB960	CTCACTGATTAAGCATTGGCACACTAACCAGCTTAGCTATGATC	GCTAAGCTGGTTAGTGTGCCAATGCTTAATCAGTGAGGC
pβ-lac-GG	Used pβ-lac and WT pVesB as template, pMMB960	TCACTGATTAAGCATTGGTCGCCTTTTGCCCCAAG	GGGCAAAAGGCGACCAATGCTTAATCAGTGAGGC
pβ-lac-IG/GG (2)	Used β-lac and WT VesB as template, pMMB960		
pVesB (3)	WT VesB	CTCACTGATTAAGCATTGGCACACTAACCAGCTTAGCTATGATC	GCTAAGCTGGTTAGTGTGCCAATGCTTAATCAGTGAGGC
pVesBGGVesA	Used pVesB and pVesA as template	GGAGGAGGTGTCTCTTTGCTTATTGCTTTC	ACCTCCTCCAGAAGAGGCACTTGG
pVesBGGVesC	Used pVesB and pVesC as template	ACCTAGGCTACCACCGCCAGAAGAGGCACTTGGCGA	TCTTCTGGCGGTGGTAGCCTAGGTGGGGCAGCCTTA
pVesBGGVC1485	Used pVesB and pVC1485 as template	GGGGGTAGCTTCGGATTAGGG	GCTACCCCCAGAAGAGGCACT
pVC1485	Used N16961 as template, VC1485 cloned into pMMB67	CAGCGGATCCACAGGAAAGTGAATAGAA	TTATCTGCAGTTGTGCCCTTCCTGACA
pVesB-VesA	Used WT pVesA and pVesB as template	TCGTATTCAACTGGATACTGGAGGGACTACAACGGTTAGC	AACCGTTGTAGTCCTCCAGTATCCAGTTGAATACGAGACGT
pVesB-VesC	Used pVesA and pVesB as template	TCGTATTCAACTGGATACTCCAAAATTCTTTGATCTACCTCCA	AGGTAGATCAAAGAATTTTGGAGTATCCAGTTGAATACGAGACGT
pVesC (3)	WT VesC		
pVesC-K508Y	Point mutant	GCCAAATAAGCTACGATCCATATTTCTTTGATCTACCTCCACC	GGTGGAGGTAGATCAAAGAAATATGGATCGTAGCTTATTTGGC
pVesC-R519Y	Point mutant	GATCTACCTCCACCGCTTGATATTTATATCGGTGATAGTGG	CCACTATCACCGATATAAATATCAAGCGGTGGAGGTAGATC
pVesAΔ23-His (2)	Used pVesA as template, phosphorylated primers	CATCACCATTGAACTTTATGATCAAGGTGTG	GTGATGGTGTCCTCCAGATGAGCTCTC
pVesAΔ23-His S221A (2)	Previously generated		
pVesBΔ20-His (2)	Used pVesB as template	CATCATCACCATCATCACTGATTACCTATCCGAGATCTG	GTGATGATGGTGATGATGACCGCCAGAAGAGGCACTTG
pVesBΔ20-His S221A (2)	Previously generated		
pVesCΔ22-His (2)	Used pVesB as template	CATCATCACTGATCTATCGATAGCCAGCATGC	GTGATGATGACCGCCGCCACTATC
pVesCΔ22-His S225A (2)	Previously generated		
pVesCΔ22-His K508Y	Used K508Y primers with pVesC Δ22-		

	His as template		
pVesA (3)	WT VesA		

Supplementary References

1. Gadwal S, Johnson TL, Remmer H, Sandkvist M. 2018. C-terminal processing of GlyGly-CTERM containing proteins by rhombosortase in Vibrio cholerae. PLoS Pathog 14:e1007341.
2. Shannon A, Johnson T, Roberts CS, Chaton CT, Korotkov KV, Sandkvist M. 2025. The PDZ domain of EpsC is required for extracellular secretion of VesB by the Type II secretion system in Vibrio cholerae. J Bacteriol doi:10.1128/jb.00144-25:e0014425.
3. Sikora AE, Zielke RA, Lawrence DA, Andrews PC, Sandkvist M. 2011. Proteomic analysis of the Vibrio cholerae type II secretome reveals new proteins, including three related serine proteases. J Biol Chem 286:16555-66.
4. Knauf VC, Nester EW. 1982. Wide host range cloning vectors: a cosmid clone bank of an Agrobacterium Ti plasmid. Plasmid 8:45-54.
5. Donnenberg MS, Kaper JB. 1991. Construction of an eae deletion mutant of enteropathogenic Escherichia coli by using a positive-selection suicide vector. Infect Immun 59:4310-7.
6. Sikora AE, Lybarger SR, Sandkvist M. 2007. Compromised outer membrane integrity in Vibrio cholerae Type II secretion mutants. J Bacteriol 189:8484-95.
7. Furste JP, Pansegrau W, Frank R, Blocker H, Scholz P, Bagdasarian M, Lanka E. 1986. Molecular cloning of the plasmid RP4 primase region in a multi-host-range tacP expression vector. Gene 48:119-31.
8. Waack U, Warnock M, Yee A, Huttinger Z, Smith S, Kumar A, Deroux A, Ginsburg D, Mobley HLT, Lawrence DA, Sandkvist M. 2018. CpaA Is a Glycan-Specific Adamalysin-like Protease Secreted by Acinetobacter baumannii That Inactivates Coagulation Factor XII. mBio 9.
9. Roberts CS, Shannon AB, Korotkov KV, Sandkvist M. 2024. Differential processing of VesB by two rhomboid proteases in Vibrio cholerae. mBio 15:e0127024.
10. Scott ME, Dossani ZY, Sandkvist M. 2001. Directed polar secretion of protease from single cells of Vibrio cholerae via the type II secretion pathway. Proc Natl Acad Sci U S A 98:13978-83.
